# Language Barriers and Timely Analgesia for Long Bone Fractures in a Pediatric Emergency Department

**DOI:** 10.5811/westjem.2020.9.48431

**Published:** 2021-01-11

**Authors:** Michelle Gaba, Hector Vazquez, Peter Homel, Antonios Likourezos, Francis See, Jess Thompson, Christine Rizkalla

**Affiliations:** * Maimonides Medical Center, Department of Emergency Medicine, Brooklyn, New York

## Abstract

**Introduction:**

Long bone fractures are common painful conditions often managed in the pediatric emergency department (PED). Delay to providing effective pediatric pain management is multifactorial. There is limited information regarding how the issue of language spoken impacts the provision of adequate and timely institution of analgesia. We sought to determine whether there is a difference between English-speaking and non-English speaking patients with respect to time to pain management for long bone fractures in a multi-ethnic urban PED.

**Methods:**

We conducted a retrospective cohort study of consecutive cases over 29 months of children <18 years old who presented to the PED with a first-time long bone fracture. A correlation of multiple clinical variables with timeliness to providing analgesia as a primary outcome was determined. We performed regression analysis to eliminate confounding and to determine the magnitude of each variable’s effect on the outcome.

**Results:**

We analyzed a total of 753 patient cases (power 0.95). Regression analysis showed that the variable of English vs non-English language spoken was the most significant predictor of timeliness to pain management (*p* < 0.001). There was a significant difference in median time to triage measurement of pain score (1 minute vs 4 minutes for English vs non-English speakers [*p* < 0.001]); median time to initial analgesia (4 minutes vs 13 minutes for English vs non-English speakers (*p* < 0.001]); and median time to opioid analgesia (32 minutes vs 115 minutes for English vs non-English speakers (*p* < 0.001]), respectively. All measurements of time were from the creation of a patient’s electronic health record. Just 30% of all patients received an opioid analgesic for treatment of long bone fractures, including only 37% with moderate triage pain scores.

**Conclusion:**

Delay to receiving analgesic medications in pediatric patients with long bone fractures can be augmented by language barriers. Time to providing analgesia for long bone fractures is significantly delayed in non-English speaking families, contributing to disproportionate care in the PED. Furthermore, use of opioid analgesia for fractures in children remains poor.

## INTRODUCTION

Pain is one of the most common complaints encountered in the emergency department (ED).^1^ Prior studies indicate that ED pain intensity is higher than in other healthcare settings, due to the higher acuity and complexity of conditions generally encountered.^2^ Despite mandatory pain assessment and safe, appropriate pain control becoming the standard of care for patients, oligoanalgesia remains a common occurrence in the ED.^3^–^6^ This problem is especially prevalent in children, even among providers who work solely in a pediatric emergency department (PED).^7^

Extremity injury is one of the more common painful conditions for which appropriate and timely management of pain is important. However, a prior study of long bone injuries in children reported that just 29% of patients received analgesia and the mean time to receiving analgesia was two hours.^8^ Despite availability of opiates and procedural sedation for ED management of long bone fractures, ED providers still underutilize and delay pain medications in children.^2^,^6^,^8^ Challenges in pediatric pain management include difficulty in assessing pediatric pain; underuse of pain assessment tools; a tendency to dismiss children’s pain as fear; time and staff constraints in the busy ED environment; and concern for adverse effects or addiction to opioid analgesia.^1^–^2^,^6^

Pain is a subjective, self-reported process, which can often vary in its manifestation among children of different cultural and ethnic backgrounds.^9^–^11^ Ethnic minorities in the United States have been shown to be at higher risk of oligoanalgesia in the ED.^12^ Prior studies demonstrated that Black, Hispanic, and Asian patients of all ages receive reduced quantity and quality of analgesia and sedation care.^13^–^18^ Minority groups have similar perception of pain to others, but differences in provider assessment of their pain negatively affect their pain management.^19^ These differences exist despite cross-cultural validity existing for multiple pediatric visual pain scales.^20^ With increasing populations of families with limited English proficiency, children with significant injuries will be placed at high risk of oligoanalgesia.

To our knowledge, only one prior published study has documented an association between limited parental English proficiency and oligoanalgesia in hospitalized children. In postoperative patients, children from limited English proficiency households were less likely to receive opioid analgesia compared to children from English-proficient households with comparable pain scores.^21^, ^22^ There are currently no published studies that correlate English proficiency with time to administering ED analgesia in children. Our objective was to assess whether a difference in timeliness to receipt of pain assessment and analgesia exists among children with long bone fractures based on the patient/family’s primary language.

## METHODS

### Study Design and Setting

We conducted a retrospective cohort study, reviewing electronic health record (EHR) charts from January 2011–May 2013. This time period was chosen due a change in the PED’s triage system that was initiated on June 1, 2013. Long bone fractures were selected for study due to the common nature of this complaint, potential for inducing significant pain, and the need for rapid analgesia and general care for these conditions.

This study was conducted in an urban PED in Brooklyn, NY, with an annual patient volume of 37,500, approximately 1400 long bone fractures per year, and a high volume of families not proficient in English. Per protocol, patients were initially triaged by a nurse, who recorded vital signs and initial pain score. Triage pain score was attained via patient self-report, using the Wong-Baker FACES scale or the Numeric Pain Rating Scale for patients aged ≥6 years. For younger patients, the FLACC (face, legs, activity, crying and consolability) behavior scale was recorded. Initial analgesia, typically acetaminophen at 15 milligrams per kilogram (mg/kg) or ibuprofen at 10 mg/kg, could be given by the nurse at that time by standing order. If no analgesia was administered at triage, the treating physician in the PED could order either one of these or an opiate medication in place of or in addition to acetaminophen or ibuprofen.

**Population Health Research Capsule**

What do we already know about this issue?

*Oligoanalgesia remains a common occurrence in the emergency department (ED), especially in children, with ethnic minorities at highest risk*.

What was the research question?

Does language play a role in timeliness of pain assessment and analgesia in a pediatric ED?

What was the major finding of the study?

*Children from non-English speaking families were more likely to experience a delay in analgesia for fractures*.

How does this improve population health?

*Greater knowledge about healthcare disparities can inform future work in settings providing care for families with limited English proficiency*.

The opiate generally favored by our PED staff was intravenous morphine at 0.05–0.1mg/kg. On-site hospital interpreters are available by request for Spanish, Mandarin, Cantonese, and Russian-speaking families. The EHR was created, with vital signs and pain score documented by the triage nurse, only once an interpreter was present to assist non-English speaking families. If on-site interpretation was unavailable, phone-based interpretation was accessible for all other languages typically encountered in our PED, with language phone lines present for use both in triage and within the PED. This study was approved and conducted in accordance with guidelines from the hospital’s institutional review board.

### Data Collection and Processing

We initially screened charts for *International Classification of Diseases*, 9^th^ revision, codes corresponding to long bone fractures of both upper and lower extremities, excluding injuries to the clavicle, hands, feet, fingers and toes as these were determined as less likely to need urgent intervention for pain. We also excluded other orthopedic conditions of torus or avulsion fractures, nursemaid’s elbow and patella dislocations, as they are not typically treated with analgesia on the same sustained basis as performed with long bone fractures. Diagnosis of long bone fracture was confirmed by a positive radiograph report in the health record as read by an attending-level radiologist. We further excluded any cases for which patients had prior history of chronic medical conditions that predisposed to limb pain (sickle cell disease and osteogenesis imperfecta) and those patients with conditions who may alter pain perception or accurate communicability (developmental delay, autism). Patients who received pain medications for their acute injury prior to hospital arrival were excluded, as were patients who indicated routine use of pain medication (defined as at least weekly use of acetaminophen or an nonsteroidal anti-inflammatory drug). In addition, we excluded patients with abnormal mental status, hypotension for age, multi-organ trauma or triage status of “resuscitation,” as their serious medical conditions could potentially have precluded pain management as a primary goal. Finally, any patient who arrived without a parent or legal guardian present was excluded because of the natural delay in care that would result while attempting to obtain consent for treatment.

Data collected included information on clinical variables that could possibly affect timely ED pain management: patient demographics (patient age, gender, ethnicity, type of health insurance); and clinical or circumstantial data relevant to treatment of their extremity injury and pain (initial pain score, triage acuity level, mode of travel to ED, patient disposition, and provision of procedural sedation). Spoken language was determined as a routine part of the triage process on all patients; interpreter usage was noted where available.

The primary outcomes measured were time intervals from creation of a patient’s EHR to each of the following pain management endpoints: time to measuring initial pain score; time to administration of initial pain medication (generally, acetaminophen or ibuprofen); and time to administration of opiate pain medication. Baseline time zero was the timestamp for the creation of the EHR; each endpoint measurement of pain management was compared to this baseline. Thus, the times to administration of pain medications already included the time to measurement of the initial pain score in triage and were not sequential time periods. Timely administration of analgesic agents was the focus of study due to the importance of rapid care for long bone fractures. Secondary outcomes analyzed related to quality of ED pain management for fractures.

### Data Analysis

We conducted a retrospective health record review in accordance with criteria set forth by prior articles.^23^ Three abstractors initially reviewed the chart data and recorded information into a templated paper data recording sheet; all abstractors underwent a training process with the primary investigator, including a mutual review of 10–15 charts, to help locate both typical and alternate portions of the health record where each data point could be found. Missing data was given a unique numerical code and accounted for in the final statistical calculations. These were then transcribed into Microsoft Excel (Microsoft Corporation, Redmond, WA) by the primary author, in order to control for any inconsistencies between reviewers and create uniformity in the data. Thus, no inter-rater reliability statistics were used. None of the abstractors or primary author were blinded to the hypothesis of the study.

We performed statistical tests using Microsoft Excel and SPSS 19.0 (SPSS Inc., Chicago, IL). Analysis included descriptive statistics and chi-square test for categorical variables. Since the variable of time would likely not follow a normal distribution curve, univariate analysis of factors (age, gender, insurance type, race/ethnicity, language spoken) related to timing of analgesia used non-parametric statistics, specifically Mann-Whitney and Kruskal-Wallis tests. Finally, we performed a regression analysis to control for potential confounders.

Statistical significance was set at *p* < 0.05. To maximize accuracy, we determined a sample size allowing a power of 95% to detect a minimal difference of five minutes to pain assessment and analgesic provision (750 patients). We set a higher standard than the traditionally used 80% limit in order to capture even the slightest difference possible between the two groups; if such a small difference was present at this level of statistical significance and power, then it would certainly be present in any larger time difference that would be more clinically relevant in the ED.

## RESULTS

**Figure 1:**
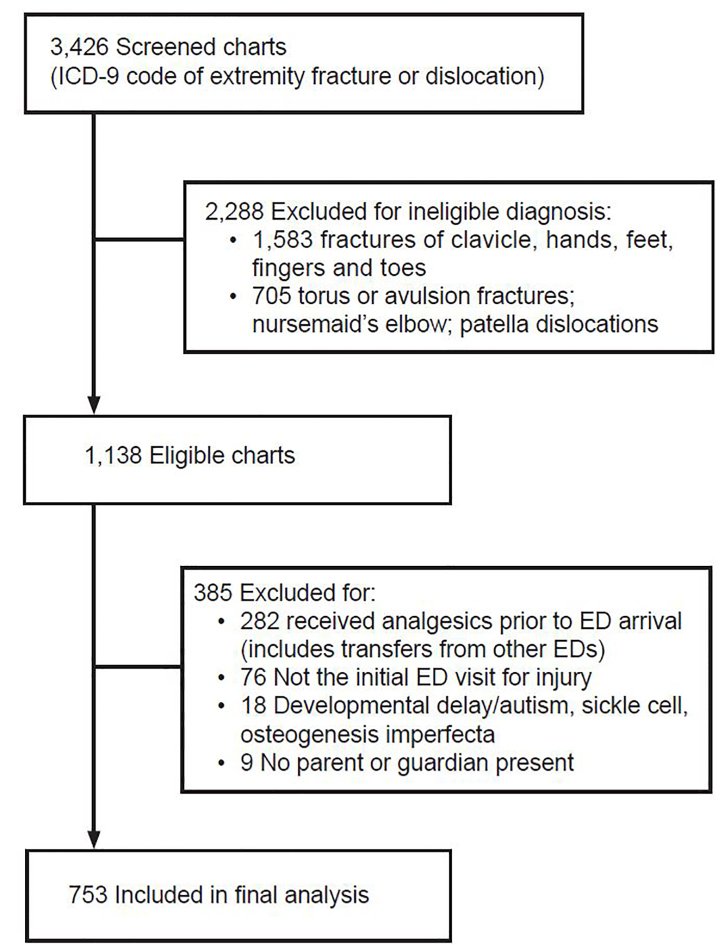
Inclusion of cases in study of pain administration to children with long bone fractures. *ED*, emergency department, *ICD-9*, Intenational Classification of Diseases, 9th revision.

We screened a total of 3426 charts for the child’s first fracture-related visit, and 753 met inclusion criteria (Figure 1). Characteristics of study subjects are detailed in Table 1. Compared to English-speaking families, patients from non-English speaking families were more likely to have Medicaid as their primary insurance. In our study population, non-English speaking families were more likely to be Hispanic or Asian, and English-speaking families were more likely to be White or Black. We initially sought to divide out each individual language spoken in our patient population: Spanish, Russian, Chinese – Mandarin and Cantonese, Urdu, Bangladeshi, Vietnamese, Yiddish, Other/Mixed. However, the variable frequencies of languages and the small number of patients who spoke each individual language were too small to analyze separately. Thus, we simplified into English and non-English groups.

The median times to initial triage measurement of pain scores, initial administration of analgesia, and opioid medication are depicted in Table 2, with each interval beginning at the creation of the patient chart in the EHR. Median time to triage measurement of pain score was 1 minute vs 4 minutes for English vs non-English speakers (*p* < 0.001); median time to initial analgesia was 4 minutes vs 13 minutes for English vs non-English speakers (*p* < 0.001); and median time to opioid analgesia was 32 minutes vs 115 minutes for English vs non-English speakers (*p* < 0.001), respectively. There was a significant difference between English vs non-English speaking patients/families in median time to triage measurement of pain score and median time to administering analgesia medications.

Secondary measures related to quality are summarized in Table 3. There was no overall difference between English and non-English speakers with respect to receipt of pain score, receipt of initial analgesia, receipt of opioid analgesia, or rate of providing procedural sedation for fracture reduction. Although there was no significant difference in opiate analgesia received between English vs non-English speaking groups, there was a significantly greater rate of an opiate administered as the initial pain mediation in the English-speaking group. In addition, among all patients who received a pain score > 0, 40 of 513 patients (8%) did not receive any pain medication; for patients with a pain score of ≥ 4, just 151 of 405 patients (37%) received an opiate medication. Overall, 198 of 661 patients (30%) who received analgesia were given opiates for long bone injuries.

Factors independently associated with enhanced timeliness to pain management included having commercial insurance, non-Asian ethnicity, non-Hispanic ethnicity, and race identified as White. The results of the linear regression show that English-speaking language was ultimately the most significant predictor of timeliness to pain management [R^2^: 0.054–0.178, *p* < 0.001].

## DISCUSSION

Providing analgesia for children is important to help relieve suffering, decrease anxiety, facilitate success in examination and diagnosis, increase patient satisfaction, and to avoid long-term neuro-psychological sequelae to painful stimuli.^21^ This study advances current general knowledge about cultural disparities in delivery of healthcare. Specifically, prior studies have outlined differences attributed to racial and socioeconomic differences, with minority children overwhelmingly more likely to receive less timely and appropriate analgesia overall in the ED.^8^ While the single-site location of this study somewhat limits its generalizability to the rest of the United States, this PED is located in one of the most diverse communities in New York City, allowing some generalizability to similar urban areas. Also, non-English speaking families seek care in many PEDs throughout the United States.

This is the first known study to address language-related barriers causing delays to analgesia administration in the PED. Among non-English speakers, there was an additional nine minutes of median wait times for initial analgesia in our ED and an additional 83 minutes of median wait times for opioid analgesia. This disparity existed despite a lack of significant difference between the two groups in terms of actual receipt of pain scores, initial analgesia, opioid analgesia, or procedural sedation for fracture or dislocation reduction. This difference was present despite the baseline time being the creation of the EHR, which would not be done for non-English speakers until there was an interpreter available to assist the patient or their family. Hence, our study design would not capture any additional delay in first obtaining an interpreter to start the registration process.

Interestingly, the one disparity in quality of pain management that existed in our study was that English speakers were more likely to receive an opiate as their first-line pain medication. It is possible that language barriers between patient and providers make it more likely that English-speaking patients would more effectively communicate degree of pain to ED providers. Also, at the time of this study, there was no intranasal formulation for rapid-onset opioid pain relief available in this institution. The extra step of explaining placement of an intravenous line for medication administration to non-English patients’ families may have been perceived as an additional barrier for staff, especially if the interpreter would need to be recalled to the ED to explain this procedure. We found no correlation between higher level of triage acuity, older patient age, or higher initial pain score with respect to timing to pain score, analgesia, or opioid analgesia.

There were some demographic differences between the two groups, with non-English speaking families more likely to have Medicaid insurance and be of Hispanic or Asian ethnicity. These differences were also independently associated with delays in pain management, which support prior studies that demonstrate minority ethnicity and Medicaid patients have less optimal pain management compared to their White and commercially-insured counterparts.^13^–^18^ However, our linear regression analysis demonstrated language spoken as the key factor in delays to pain management. With the rapid increase in diversity of the United States population and the expected increase of households with limited English proficiency, delay to analgesia may continue to compromise healthcare in these children.^21^

Overall, 92.4% of our patient population received a pain score, which parallels a prior study of long bone fractures in children and is evidence of our hospital’s standardized practice of pain assessment as mandated by the Joint Commission.^3^,^25^ Our patient population had an 87.8% rate of receipt of some form of analgesia for long bone fractures. This is a large improvement for pain management compared to prior studies that demonstrated rates of overall analgesia to be in the 30% range.^8^,^25^ We attribute this to our hospital’s protocol that allows our triage nurse to administer medications such as acetaminophen and ibuprofen to children in triage. This also allows patients to receive pain medications faster as opposed to waiting for a physician order for initial analgesia. In general, recommendations from prior pediatric studies support pain assessment along with initial triage vital signs with additional protocols to facilitate administration of acetaminophen, ibuprofen, and opiates as appropriate.^6^,^26^

One area for quality improvement in pain management is short-term acute use of opiates for long bone fractures. We found that our overall rate of opiate administration (30%) to be just as sub-par as previously documented.^8^,^16^,^25^ Attempts to educate healthcare staff about the importance of treating acute pain, the creation of national and hospital-based standards for pain management, and establishing physician-reminders to prompt analgesia order-entry have not improved rates of analgesia in children seen in the ED for painful conditions.^4^,^15^ Furthermore, in patients who received a pain score measurement of ≥ 4, just 37% received an opiate for pain control. This reflects poor compliance with guidelines and policies to use opiate medications for pain scores of ≥ 4 that other institutions follow for acute pain management.^2^,^8^

The ongoing opioid crisis in the US and concern that even legitimate pediatric exposure to opioids could be associated with subsequent abuse may be the reason that providers limit use of opiates for pain control in the scenario of long bone fractures.^7^,^27^ Alternative forms of analgesia, such as sub-dissociative doses of ketamine, or alternatives to pain medication altogether with distraction techniques, may be better suited to a given patient’s needs in acutely painful conditions and may be better used going forward given comfort with these modalities. Integrating a pain control pathway with an incremental stepwise approach to pain management that would involve the patient and their caregiver could be a future area of study.

## LIMITATIONS

The retrospective nature of the study created a reliance on accurate recordkeeping in our EHR. There was no indication in the medical record whether the provider could speak the patient’s language; thus, we did not control for any ability of staff or treating physicians to speak the patient’s native language. Future prospective studies may serve to clarify whether or not these concerns played a role in the language barriers noted by this current study. A prospective study could also explore other barriers to pain management specific to the system used in our ED, such as whether arrival during peak time is a more important factor than language issues.

Separate from the concerns that result from the retrospective nature of the study, we must acknowledge the older age of our data, which were collected from January 2011–May 2013. As noted, this time period was chosen due to a change in the ED triage process that occurred in June 2013, which would have adjusted the time intervals to the pain management variables collected for the purpose of this study. The desire to maintain uniformity in the information collected among ED staff was the primary driver of setting this cutoff date. However, we must note that major improvements in technology, access to interpreter services via video chat devices, and overall improvement in internet speed since then could yield different results had a similar study been performed in this same ED in present time. However, as a historical benchmark, this study presents important information about healthcare disparities in a non-English speaking population.

As this was a single-center study, we can only comment on the delivery of analgesia at this site alone. A prospective, multicenter study could further delineate whether language serves as a barrier to pain management and address whether this single site had any flaws in its system of administering analgesia, such as variance in triage nursing delivery of pain medication, which was the source of the differences found. Finally, further management of fractures with splinting would always occur after the initial triage process was complete, generally once the patient had already received some initial form of pain medication and radiographic studies were obtained.

As this study was focused on the initial assessment of pain and delivery of analgesia, we did not assess further means of caring for these long bone fractures and pain score reassessment. We felt that timeliness to delivery of analgesia served as a reasonable surrogate to these patient-oriented factors, despite being a more process-driven measure. Future study efforts could focus more on standardized measurements of pain score at multiple time intervals in order to assess whether the initial effect of language spoken persists throughout a patient’s ED or hospitalization course.

## CONCLUSION

Delay to providing analgesics presents a problem for non-English speaking families in the pediatric emergency department. Our study demonstrates significant delays in time to pain score (three minutes), providing initial analgesia (nine minutes), and providing opiate analgesia (83 minutes), all of which were primarily attributed to language barriers. Measures to provide translation services when needed can augment diagnostic accuracy and timeliness of patient care, as well as decrease disproportionate care to these patients. Furthermore, use of opiates overall for long bone fractures was uncommon in children, as just 30% of patients in this study received them, including 37% with moderate triage pain scores.

## Figures and Tables

**Table 1:** Characteristics of study subjects.

	English speaking (N = 369)	Non-English speaking (N = 384)	*p*-value
Median age, years	7 (4–11.5)*	6 (3–11)*	0.111
Male gender	252 (68.3%)	277 (72.1%)	0.265
Type of insurance:			
Medicaid	201 (54.9%)	299 (78.3%)	<0.001
Self-pay	22 (6.0%)	32 (8.4%)	0.258
Commercial	143 (39.1%)	51 (13.4%)	<0.001
Ethnicity/Race:			
White	214 (58.8%)	118 (31.0%)	<0.001
Hispanic	45 (12.4%)	98 (25.7%)	<0.001
Asian	29 (8.0%)	117 (30.7%)	<0.001
Middle Eastern/Indian	32 (8.8%)	21 (5.5%)	0.088
Middle Eastern (other)	29 (8.0%)	27 (7.1%)	0.678
Black	15 (4.1%)	0 (0.0%)	<0.001
Median initial pain score	5 (2–8)*	5 (2–7)*	0.023
Pain scale Used			
FACES/Numeric	203 (64.4%)	179 (55.4%)	0.024
FLACC	112 (35.6%)	144 (44.6%)	
Admitted	38 (10.4%)	51 (13.4%)	0.216
Median triage level	3 (2–4)*	3 (2–4)*	0.849
Ambulance arrival	91 (24.7%)	90 (23.4%)	0.733

* *IQR*, interquartile range.

*FACES*, Wong-Baker FACES pain scale; *FLACC*, behavioral pain scale with face, legs, activity, cry, consolability.

**Table 2:** Timeliness of pain management in pediatric patients.

	English speaking (IQR)	Non-English speaking (IQR)	*p*-value
Median time to pain score	1 minute (0–94)	4 minutes (0–155)	< 0.001
Median time to initial analgesia	4 minutes (0–91)	13 minutes (0–891)	< 0.001
Median time to opioid analgesia	32 minutes (0–221)	115 minutes (12–423)	< 0.001

*IQR*, interquartile range.

**Table 3:** Quality of pain management variables related to language barriers.

	English speaking (N = 369)	Non-English speaking (N = 384)	*p*-value
Pain score performed in triage	335 (90.8%)	361 (94.0%)	0.100
Initial analgesia administered in triage	317 (85.9%)	344 (89.6%)	0.148
Opioid analgesia administered	87 (23.6%)	111 (28.9%)	0.099
Need for sedation	65 (17.6%)	52 (13.5%)	0.132
Opiate as initially administered analgesic	35 (40.7%)	27 (24.3%)	0.020
